# Laser Processing of YSZ Ceramics without Cracking: The Impact of Solvent Selection on Performance

**DOI:** 10.1021/acsomega.4c10556

**Published:** 2025-03-18

**Authors:** Muhammad Usman, Ken-ichi Katsumata, Tetsuya Yamada

**Affiliations:** † Future Interdisciplinary Research of Science and Technology, Institute of Innovative Research, Tokyo Institute of Technology, 4259-R2-23 Nagatsuta-cho, Midori-ku, Yokohama-shi, Kanagawa 226-8503, Japan; ‡ Department of Material Science and Technology, Tokyo University of Science, 6-3-1 Niijuku, Katsushika-ku, Tokyo 125-8585, Japan

## Abstract

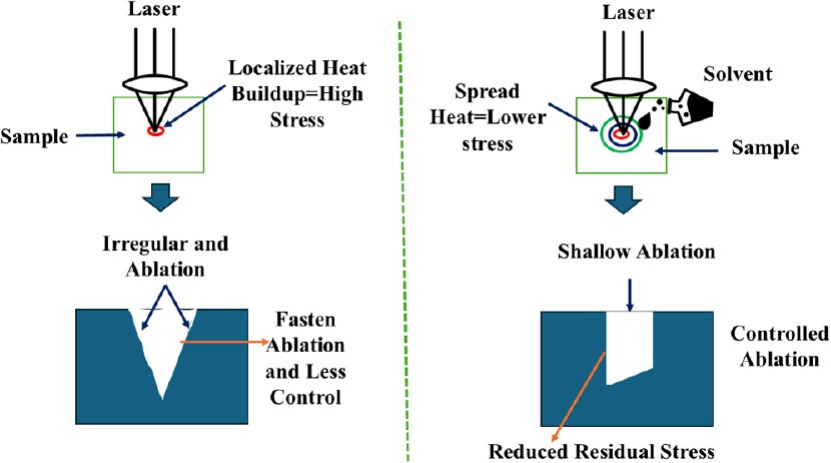

Efficient thermal management during the laser processing of ceramics is critical to prevent surface damage and maintain structural integrity, especially for materials like Yttria-stabilized zirconia (YSZ), widely used in energy, aerospace, and medical applications. However, the laser processing of YSZ ceramics poses significant challenges related to thermal stress, leading to surface damage and compromised structural performance. This study examines the effects of various cooling media, including water, acetate, and citrate acids, on the surface quality, residual stress, and ablation depth of YSZ ceramics during UV laser processing. Results indicate that acids are the most effective cooling agents, achieving minimal cracking and superior surface quality compared with other conditions. Acid-based cooling reduced the residual stress by up to 84%, enabling shallow and consistent ablation depths. These findings underscore the importance of optimized thermal management to enhance the YSZ processing quality. Potential applications extend to dental ceramics, fuel cells, and aerospace thermal barrier coatings, where enhanced surface integrity is crucial. Future studies should further explore chemical interactions between acid cooling mediums and YSZ to refine laser processing techniques for high-performance applications across industries.

## 1 Introduction

Yttria-stabilized zirconia (YSZ) ceramics are renowned for their exceptional properties, including high thermal stability, excellent ionic conductivity, robust mechanical strength, chemical inertness, and corrosion resistance, making them indispensable in advanced technological applications.^1−3^ As a key electrolyte in solid oxide fuel cells (SOFCs), YSZ facilitates efficient oxygen ion transport at elevated temperatures, which is critical for performance.^4,5^ Its superior thermal resistance and low thermal conductivity also render it essential in thermal barrier coatings for the aerospace and gas turbine industries. Moreover, YSZ’s biocompatibility and wear resistance have expanded its use in medical applications, such as dental implants and crowns, where reliability and tissue compatibility are vital.^6−10^ Beyond these, YSZ finds applications in refractory materials, wear-resistant components, and precision instruments, particularly in harsh environments like ceramic ball valves for acidic or alkaline solutions.^3,11,12^

Advancements in technology have increased the demand for precise machining of YSZ components, particularly in SOFCs and high-performance dental prosthetics, despite YSZ’s inherent hardness and brittleness.^13−15^ Traditional machining methods, including grinding, ultrasonic machining, and electrical discharge machining (EDM), are effective but face limitations. Grinding with diamond wheels achieves precise tolerances but is time-intensive, causes tool wear, and risks microcrack formation.^16^ Ultrasonic machining reduces surface damage but suffers from low material removal rates and tool wear.^17,18^ EDM requires careful control to avoid thermal damage and recast layer formation.^19^ Abrasive water jet cutting is effective for rough cuts but often necessitates additional finishing processes.^20^

Laser processing has emerged as a promising alternative for machining YSZ ceramics, offering precision, reduced processing times, and superior control over surface quality.^21^ However, YSZ’s high melting point and low thermal conductivity result in steep thermal gradients during laser processing, inducing thermal stress that promotes crack formation and compromises material integrity.^8,22,23^ Additionally, the laser-induced tetragonal-to-monoclinic phase transformation causes a 3–5% volume expansion, exacerbating internal stresses and promoting further cracking.^24^ These microcracks not only compromise structural integrity but also reduce efficiency and lifespan in critical applications like SOFCs and thermal barrier coatings.^8,25^ Moreover, they accelerate aging and low-temperature degradation, further weakening the material.^22,26^

While methods such as controlled cooling, preheating, and ultrashort pulse lasers have been explored to mitigate these issues, they often involve trade-offs between cost, complexity, and scalability. For instance, preheating reduces cracking but increases processing time, while ultrashort pulse lasers are prohibitively expensive for large-scale applications.^27−29^

Solvent-assisted laser processing has gained attention as an innovative approach to addressing these challenges. By acting as a thermal sink, solvents dissipate heat and reduce temperature gradients, thereby minimizing thermal shock and cracking. Water-assisted laser cutting, for example, has been shown to reduce the heat-affected zone by up to 40% compared to dry cutting.^30−32^ Furthermore, solvents can enhance energy deposition control by suppressing laser-induced plasma expansion, improving material removal rates, and reducing microcracking.^33,34^ However, the choice of solvent must balance the cooling efficiency with environmental and safety considerations, such as toxicity, flammability, and handling costs.

This study investigates the effects of various solvent environments, including water, acetate, and citrate acids, on the laser processing of YSZ ceramics to mitigate cracking and enhance surface quality. By comparing these solvents and a solvent-free medium, this research aims to optimize laser machining techniques for improved material integrity and performance. The findings are expected to contribute to advanced YSZ applications in industries such as energy, aerospace, and healthcare while addressing environmental and safety concerns associated with traditional processing methods.

## 2 Experiment

### 2.1 Preparation of YSZ Disk by Pressing

A 4 g weight of YSZ powder (TZ-3Y, Tosoh, Japan) was subjected to uniaxial pressing in a 67 mm diameter hardened steel die under a pressure of 35 MPa to form green disks. Due to the shrinkage inherent in ceramic sintering, the dimensions of the green disk significantly reduced after sintering. The final sintered pellet had a diameter of approximately 47 mm and a thickness of about 0.381 mm.

The sintering process of the pressed ceramics was carried out in three well-defined stages to ensure effective densification while minimizing defects. Initially, the green disk was subjected to a presintering process at 700 °C for 2 h with a slow heating rate of 0.8 °C/min. This controlled ramping rate was necessary to ensure the complete removal of organic binders without coking or introducing residual carbon impurities. The main sintering stage was performed at 1350 °C for 2 h with a higher heating rate of 2 °C/min to facilitate the densification of the YSZ structure. The density of the fully sintered YSZ pellet at 1350 °C was determined to be 6.06 g/cm^3^, confirming successful densification.

Subsequently, annealing was performed by gradually reducing the temperature from 1350 to 1000 °C over a span of 1 h. This controlled cooling process mitigated the risk of thermal shock and cracking in the YSZ material, given its brittleness and sensitivity to rapid temperature changes. The annealing process also serves to relieve residual thermal stresses and stabilize the microstructure and material properties. Following annealing, the sample was allowed to naturally cool to room temperature. The resulting YSZ disk exhibits a thickness of approximately 0.4 mm after the sintering and annealing processes. For laser ablation testing, the disk was cut into squares measuring approximately 10 × 10 mm.

### 2.2 Laser Ablation of YSZ Disk

**Figure 1:**
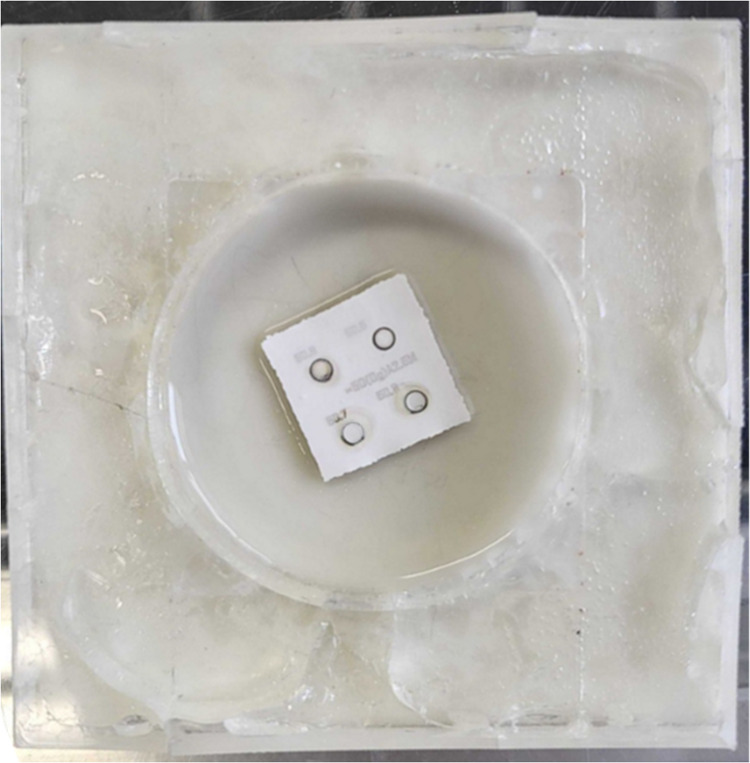
Ceramic sample placed in a 60 mm diameter well filled with a cooling medium (water, acetate, or citrate).

For the laser ablation process, a YAG laser (ALUV 5W, Akon laser, Japan) with a wavelength of 355 nm and a pulse duration of 10 ns was employed to create microscale textured grooves on the sintered ceramics. The YSZ samples were positioned on the center of the well with 60 mm diameter (Figure 1). The experiments were conducted by using a variety of solvents, including water, 2.5 M acetic acid, and 2.5 M citric acid. The pH values of these solvents were found to be 7.0, 2.0, and 0.9, respectively. These solvents were poured into the well to cover the YSZ sample. This experimental configuration allowed for effective heat dissipation during ablation and minimized the risk of surface damage. The setup was intended to manage thermal effects and to examine the impact of medium-specific cooling on the ablation depth and surface morphology.

The laser was operated at varying parameters with the pulse power (P) set at 5 W, the scanning speed (S) fixed at 50 mm/s, and the frequency (F) fixed at 30 kHz. The ablation depth was measured using a contact-based surface profilometer (Dektak (Veeco)). The surface morphologies of the YSZ compacts were analyzed before and after laser irradiation using scanning electron microscopy (SEM, QUANTA FEG 250). Residual stress during laser processing was measured by X-ray 2D “cos α method” as reported in literature,^35^ using the X-ray analysis (Pulstec, Model: μ-X360J, Japan). This nondestructive technique uses a two-dimensional detector to capture the Debye–Scherrer ring from X-ray diffraction, enabling simultaneous analysis of normal and shear stresses by examining ring distortion.

## 3 Results and Discussion

### 3.1 Impact of Solvent on Surface Quality

**Figure 2:**
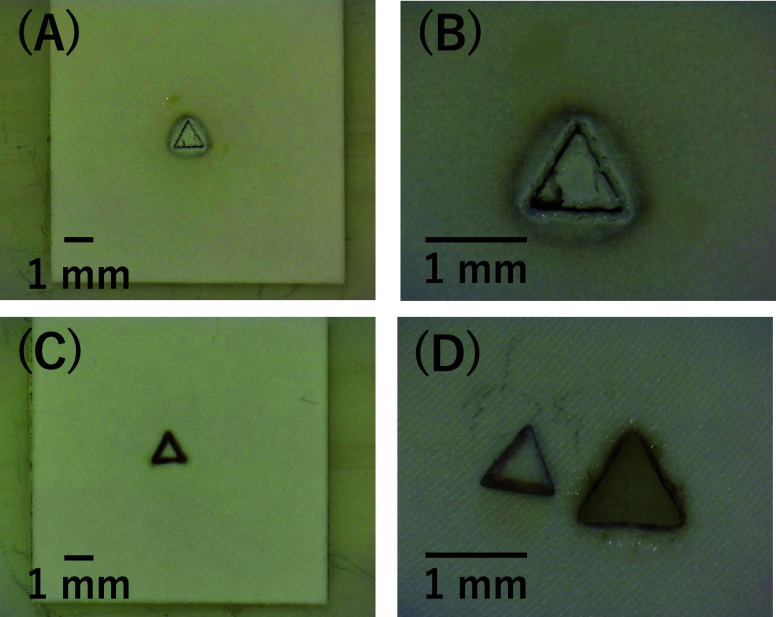
Microscopic images of YSZ post-UV laser processing without solution, low-magnification view (a) and higher-magnification view (b). Microscopic images of YSZ post-UV laser processing with 2.5 M acetate solution (c, d), showcasing the detailed surface modifications induced by the UV laser treatment. Laser Speed: 50 mm/s, number of cycle: 70–120.

The comparison between UV laser processing of YSZ with and without the use of a 2.5 M acetate solution reveals significant differences in surface modifications due to variations in thermal stress and heat dissipation. In dry processing, YSZ experiences rapid localized heating because of the high-energy laser. Given the material’s low thermal conductivity and high melting point, this leads to sharp temperature gradients, inducing thermal stresses that result in surface cracking. These cracks, which are visible in both low- and high-magnification images (Figure 2a,b), are primarily due to the insufficient heat dissipation during laser irradiation. Furthermore, the elevated temperatures induced by laser irradiation can trigger a tetragonal-to-monoclinic phase transformation in YSZ, primarily due to rapid heating and subsequent cooling.^36^ This phase transition is accompanied by a volumetric expansion of 3–5%, generating internal stresses that exacerbate the formation of microcracks. These cracks compromise the material’s structural integrity and accelerate surface degradation, reducing the reliability and performance of YSZ in high-temperature and humid environments.^37^

On the other hand, when a 2.5 M acetate solution is introduced during laser processing, the solution acts as a thermal buffer, effectively dissipating heat and reducing the temperature gradients responsible for crack formation, as reported in another study about molten salt corrosion resistance.^38^ This cooling mechanism minimizes thermal stress and helps maintain the structural integrity of the material, producing a smoother surface with fewer visible cracks, as can be seen in Figure 2c,d. The acetate solution may also chemically interact with YSZ during UV laser irradiation, potentially modifying the surface in a more controlled and uniform manner. Furthermore, the presence of the solution aids in laser-induced plasma shielding, decreasing the excess energy imparted to the surface and improving the precision of material removal.^39^ Consequently, the samples processed with the acetate solution demonstrate a markedly improved surface quality compared with those processed under dry conditions.

### 3.2 Residual Stress

**Figure 3:**
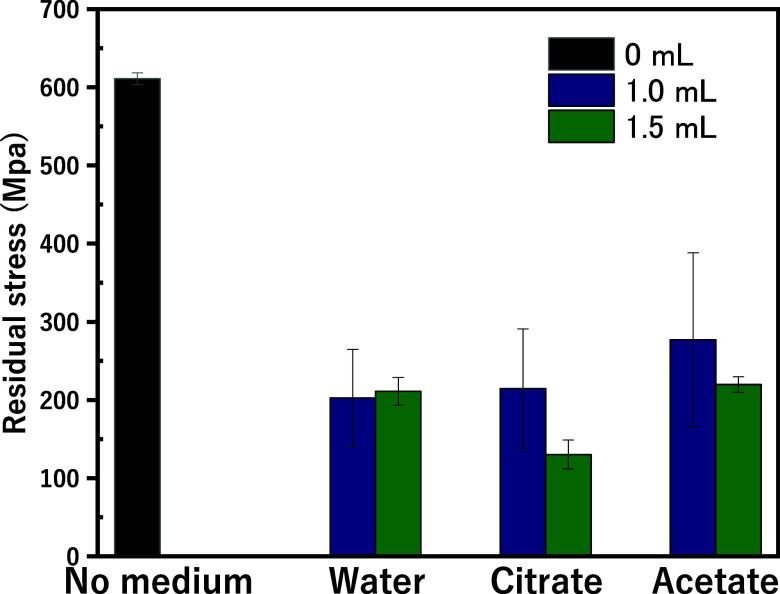
Residual stress in YSZ ceramics after UV laser processing with varying mediums.

Figure 3 illustrates the effect of different cooling mediums on the residual stress of YSZ ceramics after UV laser processing, offering critical insights into heat management during machining. Without any cooling medium (no medium), residual stress exceeds 600 MPa, indicating substantial thermal stress due to rapid localized heating and inefficient heat dissipation. This results in steep thermal gradients that create significant internal stress and potential surface cracking.^38,40^

Conversely, the introduction of cooling solutions, such as water, citrate, and acetate, dramatically reduces residual stress by 50 to 84%, depending on the solvent type and volume. Both 1.0 and 1.5 mL volumes of these solutions achieve notable stress reduction compared to dry processing, with citrate at 1.5 mL resulting in the lowest residual stress (∼100 MPa). This demonstrates citrate’s superior thermal buffering capacity, attributed to its high specific heat capacity and thermal conductivity, which allow it to absorb and dissipate thermal energy efficiently. This even heat distribution prevents the steep temperature gradients responsible for stress buildup and minimizes thermal shock, a common cause of cracking in brittle materials like YSZ.^40,41^

Additionally, citrate and acetate may interact chemically with the YSZ surface, potentially stabilizing it and preventing phase transformations. These solvents also enhance precision during machining by contributing to laser-induced plasma shielding, which reduces the total energy delivered to the material’s surface. This combination of thermal management, chemical interaction, and plasma shielding results in a significant reduction in residual stress, improving the structural integrity and durability of YSZ ceramics for high-performance applications.^42^

### 3.3 Ablation Depth and Shape

**Figure 4:**
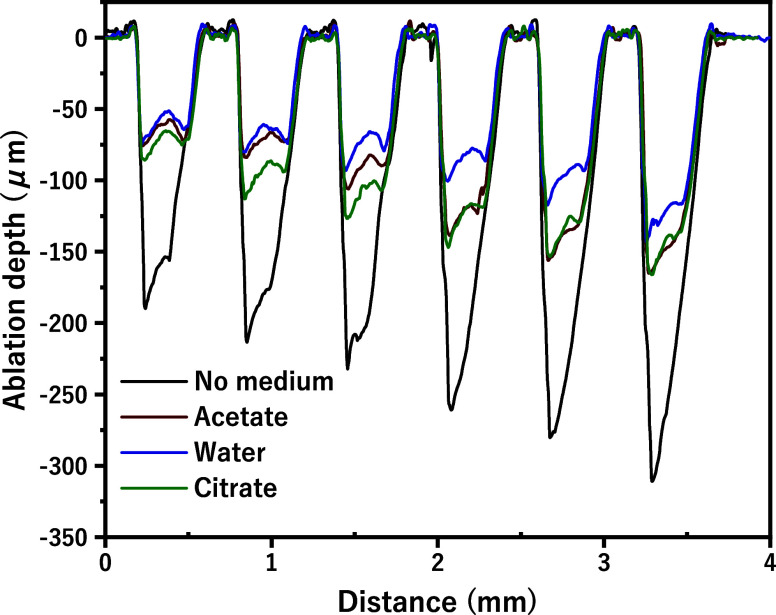
Ablation depth across a 4 mm distance for YSZ ceramics by using different mediums.

Effective shape and speed management are critical factors in the UV laser processing of YSZ ceramics, as the shape and depth of material ablation directly impact surface quality and structural integrity.^38^ To evaluate the ablation depth and shape, a rectangular region (X, 0.4 mm; Y, 2 mm) was irradiated. The laser scanning was performed with a line distance of 0.01 mm along the Y-axis direction. The irradiation was repeated 70, 80, 90, 100, 110, and 120 times for each rectangular pattern, which were arranged horizontally. The resulting patterns were measured using a contact-based surface profilometer to evaluate the ablation depth and shape along the X axis. According to the irradiation conditions, the optimum shape is rectangular grooves. Figure 4 presents the ablation depth and shape using different cooling mediums: no medium, water, and acid mediums. Each medium influences the ablation shape and processing efficiency, as observed in the material removal patterns.

Without a cooling medium (black line), the ablation depth exceeded ∼300 μm, indicating substantial material removal with an uncontrolled and irregular ablation shape. This lack of cooling resulted in rapid heating, which compromised the surface structure and produced nonoptimal shapes. The acetate medium (red line) reduced ablation depth to a range of ∼75 to ∼160 μm compared to the no-medium condition, but its shape remained less controlled, suggesting that acetate provides only moderate shape stability and slows processing speed moderately. Water (blue line) yielded improved control with a shallower ablation depth (∼75 to ∼150 μm) and a more uniform shape. With its high specific heat capacity, water dissipated heat more effectively, creating a stable environment that led to a smoother ablation profile. The citrate medium (green line) provided the shallowest and most consistent ablation depth (∼80 to ∼160 μm), achieving the most optimal shape (rectangular grooves). Achieving an optimal ablation shape that is both shallow and consistent not only minimizes surface damage but also enhances the precision required for high-performance applications. This efficiency in managing shape and depth suggests that the acidic medium may chemically stabilize the YSZ surface, further supporting precise laser processing.

**Figure 5:**
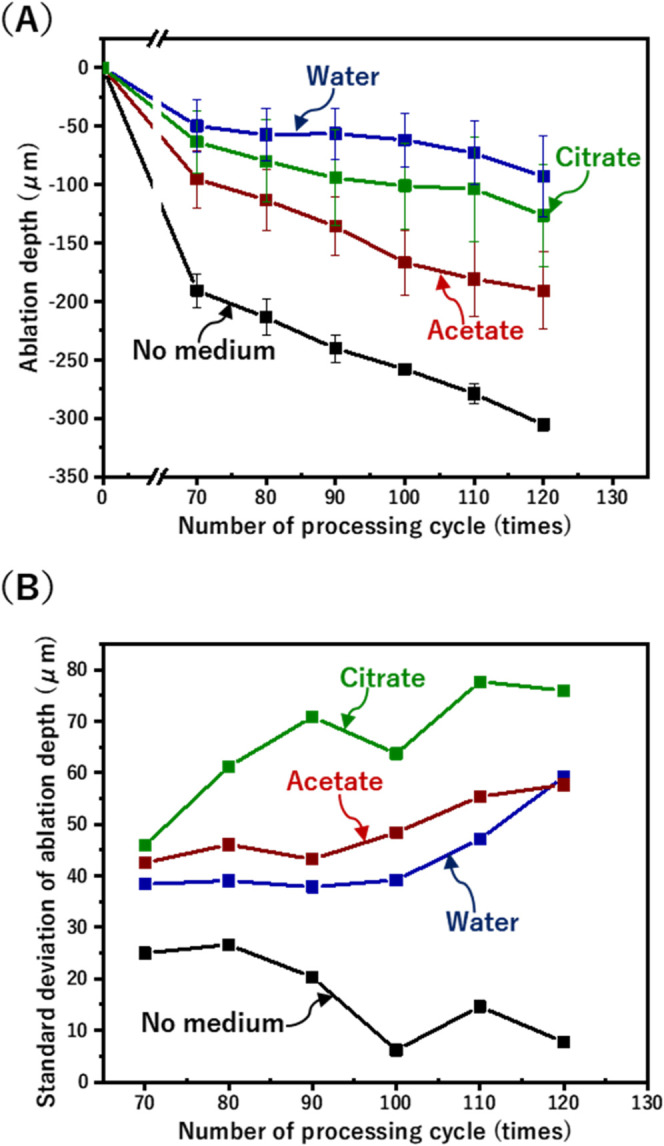
(a) Ablation depth and (b) standard deviation of ablation depth vs number of processing cycles for various mediums.

Figure 5a illustrates the evolution of ablation depth over multiple laser cycles for each medium, highlighting how acidic media influence ablation effectiveness. The “no medium” condition exhibited a rapid increase in ablation depth due to progressive material damage from accumulated heat. Acetate demonstrated a more moderate depth increase, though its overall ablation depth remained higher than that of water and citrate, indicating its limited thermal management capabilities. Water maintained a stable ablation depth over cycles, reflecting consistent thermal regulation. Acidic media, such as acetate and citrate, facilitate efficient ablation, with citrate exhibiting the shallowest and most uniform depth over multiple cycles.

The observed trends suggest that acidic mediums enhance ablation through both thermal and chemical interactions with the YSZ surface, reducing the variability during laser processing. Figure 5b depicts the standard deviation of the ablation depth, showcasing differences in the stability of material removal. The “no medium” condition produced deep ablation, indicative of inefficient heat dissipation. Acetate displayed moderate fluctuations in depth, while water ensured stable and uniform ablation with minimal variability. Citrate showed slightly higher variability in the initial cycles but quickly stabilized, supporting consistent material removal. The chemical and thermal buffering properties of acidic mediums thus contribute to promoting efficient ablation, even in nonprecise, cycle-based scenarios.

**Figure 6:**
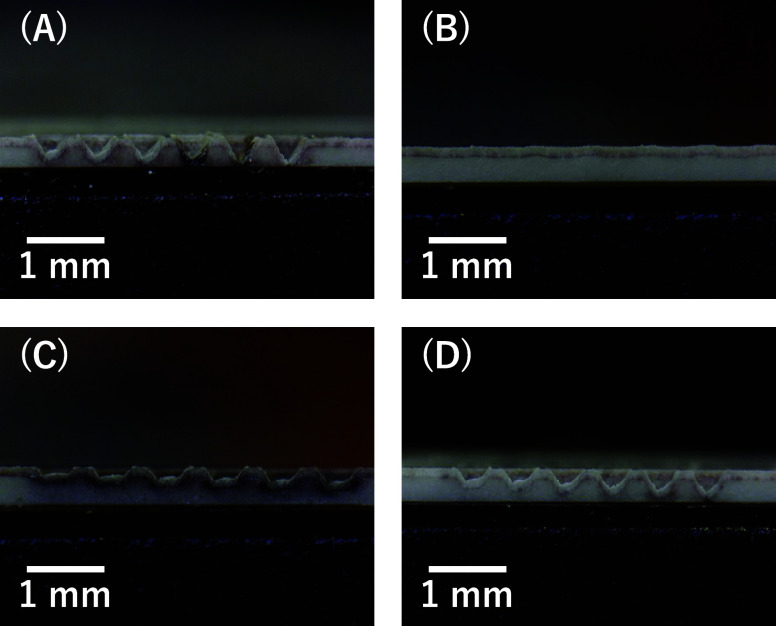
Side view images of YSZ surfaces after UV laser processing with different mediums: (A) no medium, (B) water, (C) citrate, and (D) acetate.

In addition, Figure 6 presents microscopic images of YSZ surfaces after UV laser processing using different cooling mediums: no medium, water, citrate, and acetate.

The surface processed without a medium (A) showed significant roughness and irregularities (close to V-shaped groove), indicating severe thermal damage, which corresponds to the deepest ablation observed in Figure 4. In contrast, water (B) resulted in not deep ablation, as seen in Figures 4 and 5. Citrate (C) facilitates the formation of well-defined rectangular grooves, achieving an optimal shape with minimal surface irregularities. Additionally, the grooves’ ablation depth increases consistently with the number of laser irradiation cycles, highlighting a clear relationship between the processing parameters and the resulting groove dimensions. These observations confirm the effectiveness of citrate in enhancing both the precision and uniformity of laser ablation on the YSZ surface. Acetate (D) showed a rectangular groove though slightly less effective than citrate. These observations visually confirm the findings from Figures 4 and 5, demonstrating that cooling mediums such as citrate and acetate not only promote ablation depth compared to water but also significantly improve surface quality by managing thermal stress more effectively during the laser processing of YSZ ceramics.

### 3.4 SEM Results

**Figure 7:**
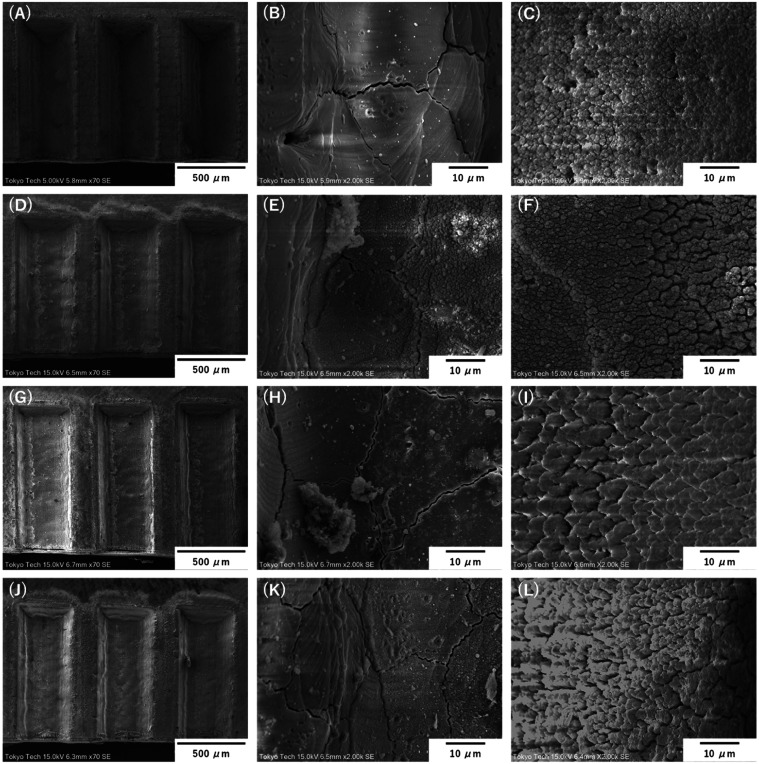
SEM images of YSZ surfaces post-UV laser processing using different mediums such as no medium (A–C), acetate (D–F), water (G–I), and citrate (J–L).

The SEM images in Figure 7 offer a detailed examination of the surface morphology of YSZ ceramics following UV laser processing with various cooling mediums: no medium, acetate, water, and citrate. The images, captured at magnifications of 50 μm (A, D, G, J) and 10 μm (B, C, E, F, H, I, K, L) using SEM at 15.0 kV, working at a 6.5 mm distance with an SE detector (x70), provide valuable insights into the impact of these mediums on surface quality.

Beginning with the no-medium condition, the surfaces show significant signs of thermal damage. The deep grooves and rough, irregular edges seen in Figure 7A indicate substantial material removal due to the lack of effective heat dissipation. This thermal stress is even more evident in Figure 7B, where large cracks and pronounced surface irregularities dominate, highlighting the material’s inability to cope with the generated heat. Finally, Figure 7C presents a highly textured surface with extensive cracking, further confirming the severe degradation that occurs in the absence of a cooling medium. Shifting to the acetate medium, improvements in thermal management are apparent. In Figure 7D, the grooves appeared smoother compared to the no-medium condition, suggesting that acetate offers some level of heat control. This improvement is more evident in Figure 7E, where reduced cracking and more uniform surface features indicate that acetate helps to buffer thermal stress. However, its effectiveness remains limited compared to water or citrate. Figure 7F shows a relatively smooth surface with minimal defects, highlighting acetate’s moderate success in cooling performance.

When water is used as the cooling medium, further enhancements in the surface quality are observed. Figure 7G shows moderately smooth grooves, reflecting significant improvement in heat dissipation compared to the no-medium condition. Surface damage is less severe in Figure 7H, with some visible cracking, although much less pronounced than in previous conditions. Figure 7I reveals a uniform surface with fewer defects, underscoring water’s capability for consistent and reliable thermal management during the process. Finally, the citrate medium produced the best results in terms of surface quality. Figure 7J exhibits the smoothest grooves among all of the conditions, indicating superior control over the thermal environment. This is further supported by Figure 7K, which shows slightly reduced cracking and a highly uniform surface, highlighting citrate’s remarkable efficiency in minimizing thermal stress. Figure 7L presents the least textured surface with almost no defects, conclusively demonstrating citrate’s effectiveness in enhancing surface quality during the UV laser processing of YSZ ceramics.

These SEM results, consistent with findings from Figures 4–6, clearly demonstrate that citrate provides the most effective thermal management, resulting in smoother surfaces, minimal cracking, and fewer defects. Water also proves to be an efficient cooling medium, while acetate offers moderate improvements over the no-medium condition. Together, the SEM images and quantitative data provide compelling evidence that optimizing thermal management significantly enhances the surface integrity during the ablation process.

## 4 Conclusions

This study demonstrated the significant role of cooling mediums in the UV laser processing of YSZ ceramics. The introduction of acetate and citrate solutions notably improved surface quality, minimized residual stress, and enhanced the machining performance compared to other cooling agents. Specifically, acid-based cooling achieved a reduction in residual stress of up to 84%, resulting in shallow and consistent ablation depths, while minimizing surface cracking. These findings underscore the importance of optimized thermal management during laser processing to maintain the structural integrity and surface quality of the YSZ ceramics. The findings may have broad implications for industries such as aerospace, automotive, semiconductor manufacturing, dental ceramics, fuel cells, and thermal barrier coatings, where precision, material resilience, and surface integrity are critical. Further investigation into the chemical interactions between acid-based cooling media and YSZ is recommended to refine laser processing techniques for high-performance applications in various sectors.
